# Galectin-3 protects distal convoluted tubules in rhabdomyolysis-induced kidney injury

**DOI:** 10.1007/s00424-024-02987-0

**Published:** 2024-07-23

**Authors:** Vera A. Kulow, Robert Labes, Claudia S. Czopek, Christian Rosenberger, Michael Fähling

**Affiliations:** 1 Institut für Translationale Physiologie (CCM), Charité–Universitätsmedizin Berlin, corporate member of Freie Universität Berlin and Humboldt-Universität zu Berlin, Charitéplatz 1, 10117 Berlin, Germany; 2 Medizinische Klinik m.S. Nephrologie und Internistische Intensivmedizin (CCM), Charité–Universitätsmedizin Berlin, corporate member of Freie Universität Berlin and Humboldt-Universität zu Berlin, Charitéplatz 1, 10117 Berlin, Germany

**Keywords:** AKI, Advanced glycation end products, Rhabdomyolysis, Lgals3, Apoptosis

## Abstract

Advanced glycation endproducts (AGEs) contribute to cellular damage of various pathologies, including kidney diseases. Acute kidney injury (AKI) represents a syndrome seldom characterized by a single, distinct pathophysiological cause. Rhabdomyolysis-induced acute kidney injury (RIAKI) constitutes roughly 15% of AKI cases, yet its underlying pathophysiology remains poorly understood. Using a murine model of RIAKI induced by muscular glycerol injection, we observed elevated levels of AGEs and the AGE receptor galectin-3 (LGALS3) in the kidney. Immunofluorescence localized LGALS3 to distal nephron segments. According to transcriptomic profiling via next-generation sequencing, RIAKI led to profound changes in kidney metabolism, oxidative stress, and inflammation. Cellular stress was evident in both proximal and distal tubules, as shown by kidney injury markers KIM-1 and NGAL. However, only proximal tubules exhibited overt damage and apoptosis, as detected by routine morphology, active Caspase-3, and TUNEL assay, respectively. In vitro, distal convoluted tubule (DCT) cells challenged with AGEs underwent apoptosis, which was markedly enhanced by *Lgals3* siRNA treatment. Thus, in RIAKI, the upregulation of LGALS3 may protect the distal nephron from AGE-mediated damage, while proximal tubules lacking LGALS3 stay at risk. Thus, stimulating LGALS3 in the proximal nephron, if achievable, may attenuate RIAKI.

**Supplementary Information:**

The online version contains supplementary material available at 10.1007/s00424-024-02987-0.

## Introduction

Advanced glycation endproducts (AGE) promote cell damage [38]. They have garnered attention due to their potential role in inflammation [2], oxidative stress [39, 49], and cellular dysfunction [49]. AGEs are complex molecules, formed by non-enzymatic reactions between reducing sugars and proteins, lipids, or nucleic acids [42]. Typically, AGEs can be produced from external sources, such as food processing at high temperatures, or endogenously through various pathways, including the Hodge pathway (resulting from the autoxidation of Amadori products), the Namiki pathway (stemming from the degradation of amino acids or lipids and the cleavage of dicarbonyl compounds from aldimines), and the Wolff pathway (which involves the formation of carbonyls following the autoxidation of monosaccharides like glucose, fructose, ribose, and glyceraldehyde) [37, 56, 57]. Methylglyoxal serves as the predominant endogenous mediator responsible for the synthesis of AGEs, found ubiquitously across all cells [42]. Accordingly, AGEs have been implicated in various pathologies, including diabetes [3, 53], cardiovascular diseases [40, 41], and chronic kidney disease [44, 55]. AGEs exert their biological effects through interactions with specific receptors, most notably RAGE, which are widely expressed in various cell types, including renal cells [8]. Next to RAGE, there are several known AGE-receptors including DDOST (OST-48, AGE-R1), PRKCSH (80 K-H, AGE-R2), and galectin-3 (LGALS3, AGE-R3) [48], which have been shown to form complexes and interact with each other. Nevertheless, their mechanistic properties and potential role in kidney diseases have not been sufficiently elucidated [53]. AGE clearance occurs in the kidney and, thus, this organ is particularly vulnerable to AGE-mediated damage [15]. In humans, the level of circulating AGEs aligns with the risk of chronic kidney disease (CKD) and all-cause mortality [26, 31]. Initial investigations into the connections between AGEs and kidney damage were also carried out in acute kidney injury caused by ischemia–reperfusion [30], representing the most popular AKI model. However, the spectrum of AKI is very variable with approximately 21 different etiologies [1]. In this spectrum, rhabdomyolysis-induced AKI (RIAKI) accounts for approximately 15% of total causes [48]. Mechanistically, myoglobin, released from damaged muscle tissue, leads to tubular obstruction and oxidative stress [19, 47], ultimately culminating in AKI [17, 19].

The transcriptomic signature of RIAKI indicates that AGEs could be an important driver of this particular form of cell damage. Indeed, we found widespread and abundant AGEs in RIAKI kidneys. AGE receptor LGALS3 was exclusively upregulated in the distal nephron, a segment protected from acute injury despite being exposed to stress. Since in vitro LGALS3 downregulation exacerbated AGE-induced apoptosis, LGALS3 may serve as a new renoprotective factor.

## Materials and methods

### Animals

Animal experiments were approved by local authorities (Landesamt für Gesundheit und Soziales, Berlin: L0206/20) and carried out in line with the guidelines of the American Physiological Society. Male C57BL/6NCrl mice (24–31 g body weight) were fed a standard rodent chow and had free access to drinking water.

### Rhabdomyolysis-induced acute kidney injury (RIAKI)

In order to induce RIAKI, drinking water was withheld for 19 h, followed by IM injection of either 50% glycerol (0.05 ml per 10 g body weight; *N* = 10) or saline (sham controls; *N* = 10) into the left hind limb under isoflurane anesthesia. For pain management, Rimadyl© (5 mg/kg body weight *i.p.*) was administered at 0 and 3 h. At 24 h, blood was obtained from the mandibular venous plexus. Mice were euthanized by cervical dislocation, and kidneys were snap-frozen or immersion-fixed in 4% paraformaldehyde for 24 h.

### Blood parameters

Plasma creatinine levels were measured by Labor Berlin—Charité Vivantes GmbH (Berlin Germany).

### Cell culture experiments

Mouse distal convoluted tubular (DCT) cells (209/MDCT; #CRL-3250; ATCC, USA; passage 3 up to 15) were used for in vitro experiments. Cells were cultured at 37 °C and 5% CO_2_, using RPMI-1640 Medium (Sigma-Aldrich, USA), supplemented with 10% (v/v) fetal bovine serum (Biochrom GA, Germany), 1% (v/v) penicillin–streptomycin (10,000 U/mL, Thermo Fisher Scientific, USA), and 1% (v/v) L-glutamine solution (200 mM, Sigma-Aldrich, USA).

*Lgals3* knockdown was achieved by transfecting cells with 25 nM of ON-TARGETplus *Lgals3* (#L-041097–01-0050, Horizon Discovery Ltd., UK) directed siRNA pool and compared to mock transfection using 25 nM ON-TARGETplus non-targeting control pool (mock, #D-001810–10-20, Horizon Discovery Ltd., UK). DharmaFECT 1 Transfection Reagent (Horizon Discovery Ltd., UK) was applied for transfection according to the manufacturer’s protocol. Briefly, siRNA pool and DharmaFECT 1 transfection reagent were diluted in serum-free medium, mixed, and incubated for 20 min at room temperature before adding to the cells.

To test the effect of AGEs, cells were treated with either 200 µg/ml AGE-BSA (Cayman Chemical, USA) or normal BSA (Carl Roth GmbH, Germany) as a control. After 24 h of transfection, cells were exposed to BSA or AGE-BSA, respectively, for another 24 h. Then, cells were harvested with RNA-STAT-60 (Tel-Test Inc., USA) for RNA isolation or used for TUNEL assay analysis.

### Quantitative PCR

RNA extraction from frozen kidney samples was performed using RNA-STAT-60 (Tel-Test, Inc. USA) according to the manufacturer’s instructions followed by cDNA synthesis with random primers and Superscript II reverse transcriptase (Thermo Fisher Scientific Inc., USA).

qPCR was performed as described in Labes et al*.* [24]. Triplicate analyses were conducted, and their mean values were normalized against *18S rRNA* using the ΔΔCt-method. Primer sequences are shown in Supplementary Table 1.

### Next-generation sequencing (NGS)

For high-throughput 3′ transcriptome analysis, RNA was extracted as described. For next-generation sequencing, the QIAGEN Genomic Services (https://www.qiagen.com/us/applications/next-generation-sequencing/genomic-services/rna-sequencing-services/mrna-ngs-seq-service) was engaged. The QIAseq UPX 3′ Transcriptome Kit (QIAGEN) was used for library preparation, converting 2.5 µl purified RNA into cDNA NGS libraries. During reverse transcription, each sample received a unique ID, and each RNA molecule was tagged with a unique molecular index (UMI). Library quality control utilized capillary electrophoresis (Agilent DNA 7500 Chip, Agilent Technologies, Inc., USA), and quantification was done using qPCR. Libraries meeting quality standards were equimolarly pooled. Sequencing was performed on a NextSeq 500 (Illumina, Inc., USA) instrument following manufacturer instructions (100 bp read length for read 1, 27 bp for read 2). Raw data was demultiplexed using bcl2fastq2 software (Illumina, Inc., USA), and FASTQ files were generated for each sample. Demultiplexed sequencing reads were processed using the “Demultiplex QIAseq UPX 3′ reads” tool of CLC Genomics Workbench 21.0.4. The “Quantify QIAseq UPX 3′ workflow” was applied with default settings, annotating reads with UMIs, trimming for poly(A) and adapters, and deduplicating based on UMIs. Reads were then mapped to the mouse genome GRCm38 v. 80 and annotated using ENSEMBL GRCm38 v. 86 gene annotation. Differential expression analysis was performed using the “Empirical analysis of DGE” algorithm of CLC Genomics Workbench 21.0.4, implementing the “Exact Test” for two-group comparisons by Robinson and Smyth [45] and incorporated in the EdgeR Bioconductor package [52]. Genes with at least 10 counts summed across all samples were considered for unsupervised analysis. Raw count matrices underwent variance stabilizing transformation using the vst function of the R package DESeq2 version 1.28.1.

### Gene set enrichment analysis

For gene set enrichment analysis (GSEA), the SetRank package [52] for R was used. The analysis was carried out according to the provided instructions (https://cran.r-project.org/web/packages/SetRank/vignettes/vignette.pdf) with the hallmark annotation tables from MSigDB [25]. All mapped genes were used as background set. For building the set collection, a maxSetSize of 500 was used. SetRank analysis was performed with ranks and a FDR cutoff of 0.01. Data were visualized using the GOplot package [59] for R.

### Morphological studies

Kidney sections were prepared from paraffin-embedded samples and processed for staining after deparaffinization and rehydration. Staining methods included immunofluorescence (IF), immunohistochemistry (IHC), periodic acid–Schiff (PAS), and TUNEL assay. Antibodies employed are listed in Supplementary Table 2. Except for PAS staining, slices were cooked in a pressure cooker (WMF, Germany) for 12 min in Target Retrieval Solution (Agilent Technologies, Inc., USA).

Specifically, for PAS staining, rehydrated slices were treated with the PAS-staining kit from Morphisto according to the manufacturer’s protocol (Cat. #12153.00500, Morphisto GmbH, Germany). Stained slices were dehydrated and mounted with a synthetic mounting medium (Roti®Histokitt II, Cat. #T160.1, Carl Roth GmbH, Germany). For IHC and IF, unspecific proteins were blocked for 1 h at room temperature with RTU horse serum (Vector Laboratories, USA) (IHC) or 5% skimmed milk in TBS-T (IF). Primary antibodies were diluted in RTU horse serum (IHC) or antibody-diluent (Agilent Technologies, Inc., USA) (IF) and incubated overnight at 4 °C, followed by incubation with an HRP conjugated secondary antibody (Vector Laboratories, USA) for 1 h at room temperature. For IHC, slices were developed with DAB (3, 3′-diaminobenzidine, Vector Laboratories, USA) under visual control. Stained slices were mounted using Immu-MountTM (Thermo Fisher Scientific, USA). Images were recorded using an Eclipse Ti2-A microscope and a DS-Ri2 camera controlled through the NIS-Elements software (Nikon, USA). To obtain large images, single images were recorded and stitched together afterward.

### TUNEL staining

For terminal deoxynucleotidyl transferase dUTP nick end labeling (TUNEL) staining, both kidney slices and adherent DCT cells were tested. Kidney slices were incubated in TUNEL solution (In Situ Cell Death Detection Kit, TMR red; Roche, Switzerland) along with the primary antibody, followed by washing steps in TBS-T and subsequent application of the secondary antibody diluted in antibody-diluent (Agilent Technologies, Inc., USA). The stained slices were mounted using Immu-Mount™ (Thermo Fisher Scientific Inc., USA). Similarly, adherent DCT cells were fixed with paraformaldehyde (4% in 1 × PBS) and permeabilized with 0.1% Triton X-100 in 0.1% sodium citrate. After washing, the TUNEL reaction mixture was added and incubated according to the manufacturer’s protocol. Hoechst 33342 (Thermo Fisher Scientific Inc., USA) was used for nuclear counterstaining, and the percentage of TUNEL-positive cells relative to the total cell count was calculated for data interpretation.

### Western blotting

Western blotting was performed as described in Labes et al*.* [24]. Membranes were incubated with primary antibodies (listed in Supplementary Table 2) at 4 °C overnight, followed by incubation with a secondary antibody for 1 h at room temperature. Protein signals were visualized using a chemiluminescence solution (WesternBright Chemilumineszenz Substrat Sirius, Biozym, Germany) and a Chemostar Imager (Intas Science Imaging Instruments GmbH, Germany). Subsequently, membranes were stripped and reprobed using a class IIb β-tubulin antibody (anti-Tubb2B, proteintechTM, #10063–2-AP), which served as a loading control.

### Quantification and statistical analysis

The GraphPad Prism software (Version 8, USA) was used for all statistical analyses. Identification of outliers was done by the ROUT method (*Q* = 5%) [32]. For the analysis of two groups, the normal distribution was tested with the Kolmogorov–Smirnov test. If data were normally distributed and equal SD could be assumed (largest SD difference < twofold), an unpaired Student’s *t* test was used. If equal SD could not be assumed (largest SD difference > twofold), Welch’s *t* test was performed. For data that did not follow a Gaussian distribution, the nonparametric Mann–Whitney test (equal SD) or Kolmogorov–Smirnov test (equal SD not assumed) was used. When comparing 4 groups, the ordinary one-way ANOVA followed by Tukey’s post-hoc test was applied. Data were visualized by boxplots with median, lower and upper quartile, and minimum and maximum range of values as whiskers. Dots represent single values. *p*-values below 0.05 were considered significant.

## Results

### Rhabdomyolysis leads to profound functional and morphological kidney damage

**Fig. 1:**
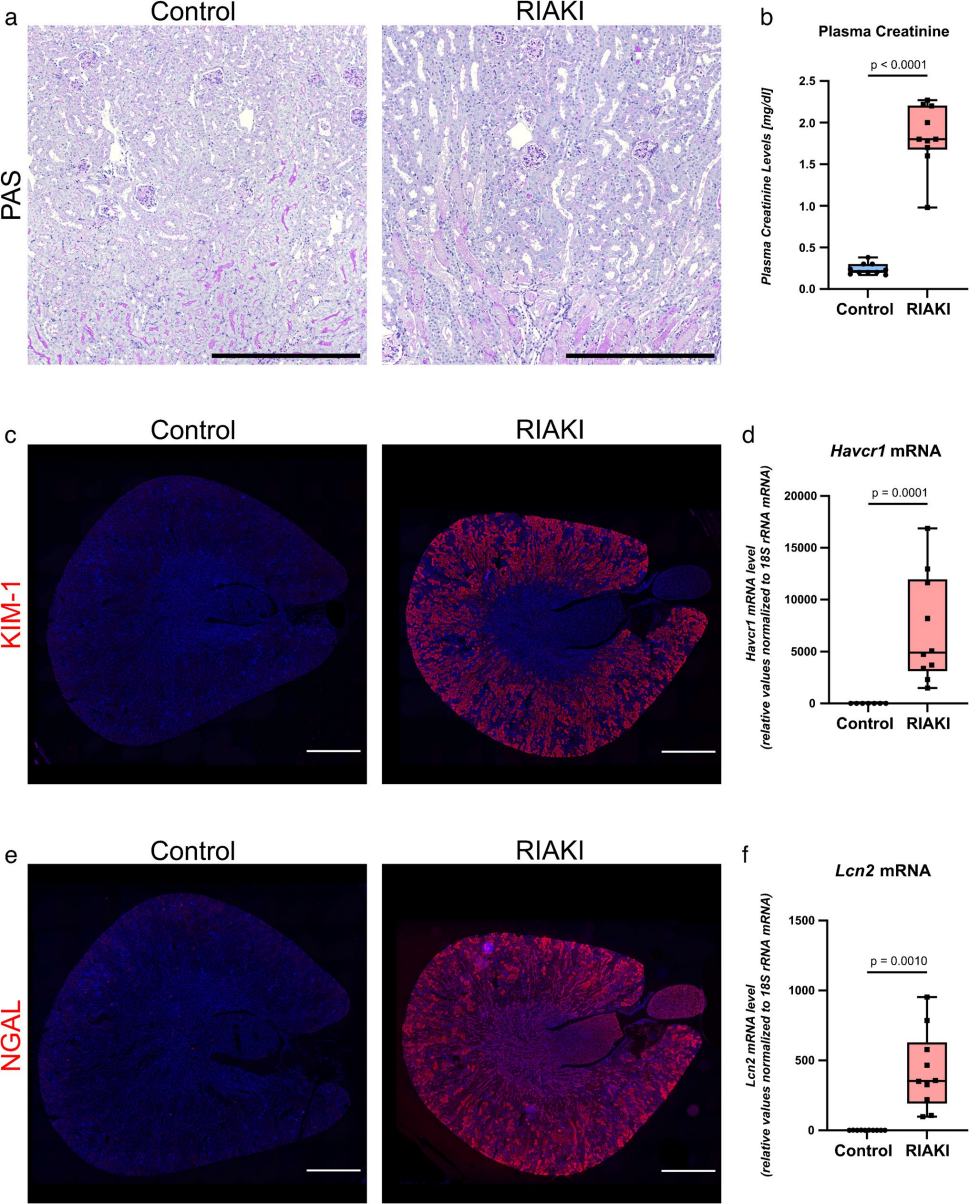
Assessment of renal injury following RIAKI (24 h). **a** Periodic acid-Schiff staining, **b** plasma creatinine; **c**, **e** immunofluorescence staining for kidney injury markers KIM-1 and NGAL in mouse kidneys 24 h after induction of RIAKI; Scale bar: 1000 µm. **d**, **f** qPCR analysis for *Havcr1* (gene for KIM-1) and *Lcn2* (gene for NGAL). RIAKI is confirmed by conventional histology, plasma creatinine, and kidney injury markers. Box plots show the median with lower and upper quartile as box. Whiskers show the minimum and maximum values. Dots represent single values. Statistical analysis was performed using either Welch’s *t* test (passed normality test with no equal SD) or Kolmogorov–Smirnov test (did not pass normality test and no equal SD). Adjusted *p*-values are shown

Twenty-four hours after induction of RIAKI [14], PAS staining revealed tubular damage such as loss of brush border, cell disruption, loss of cell integrity, tubular cast formation and necrosis (Fig. 1a, Supplementary Fig. 1a–d), plasma creatinine was elevated (Fig. 1b), and cellular injury markers KIM-1 and NGAL appeared de novo (Fig. 1c–f, Supplementary Fig. 1e, f).

**Fig. 2:**
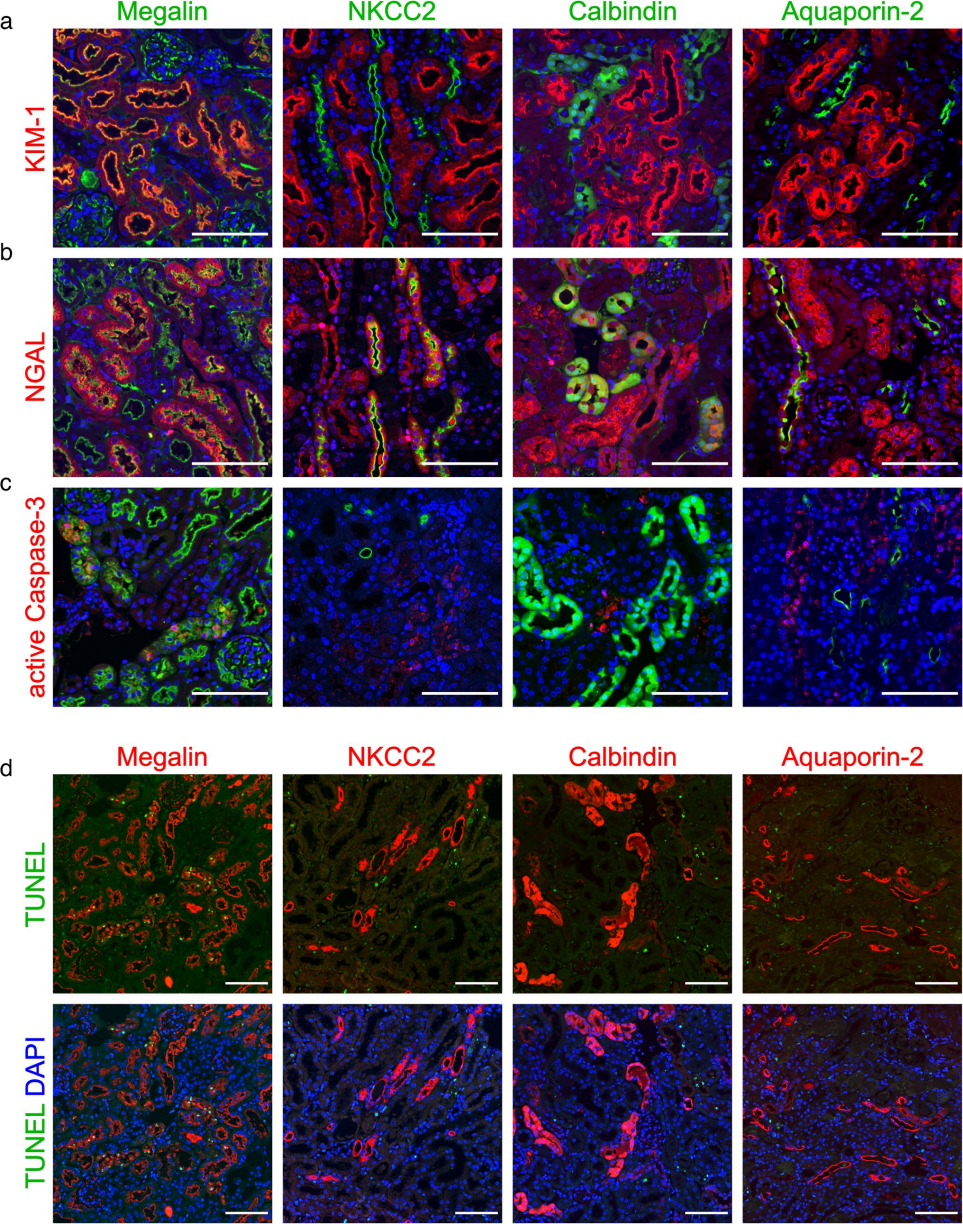
Localization of kidney injury and apoptosis in RIAKI. Immunofluorescence on mouse kidneys 24 h after induction of RIAKI. No markers for kidney injury or apoptosis were observed in controls (not shown), but prominent staining was evident in RIAKI. Double staining for nephron segment markers (green in **a** to **c**, red in **d**) megalin (PT), NKCC2 (TAL), calbindin (DCT and CNT), and aquaporin-2 (CNT and CD), respectively, with either KIM-1 (red in **a**), NGAL (red in **b**), active caspase-3 (red in **c**), or TUNEL assay (green in **d**). KIM-1, active caspase-3, and TUNEL signals appeared in PT, whereas NGAL appeared throughout the nephron. Scale bars: 100 µm

Double staining with nephron-specific markers (megalin for PT, NKCC2 for TAL, calbindin for DCT and CNT, aquaporin-2 for CNT and CD; Fig. 2) demonstrated KIM-1 exclusively in the PT, consistent with its role as a PT-specific marker [18] (Fig. 2a), while NGAL was expressed de novo throughout the nephron (Fig. 2b). However, active caspase-3 and TUNEL signals revealed apoptotic cells only in the PT (Fig. 2c, d).

In summary, rhabdomyolysis resulted in AKI within 24 h, primarily affecting the PT, as evidenced by elevated damage markers and apoptotic cells.

### The RIAKI-transcriptome

**Fig. 3:**
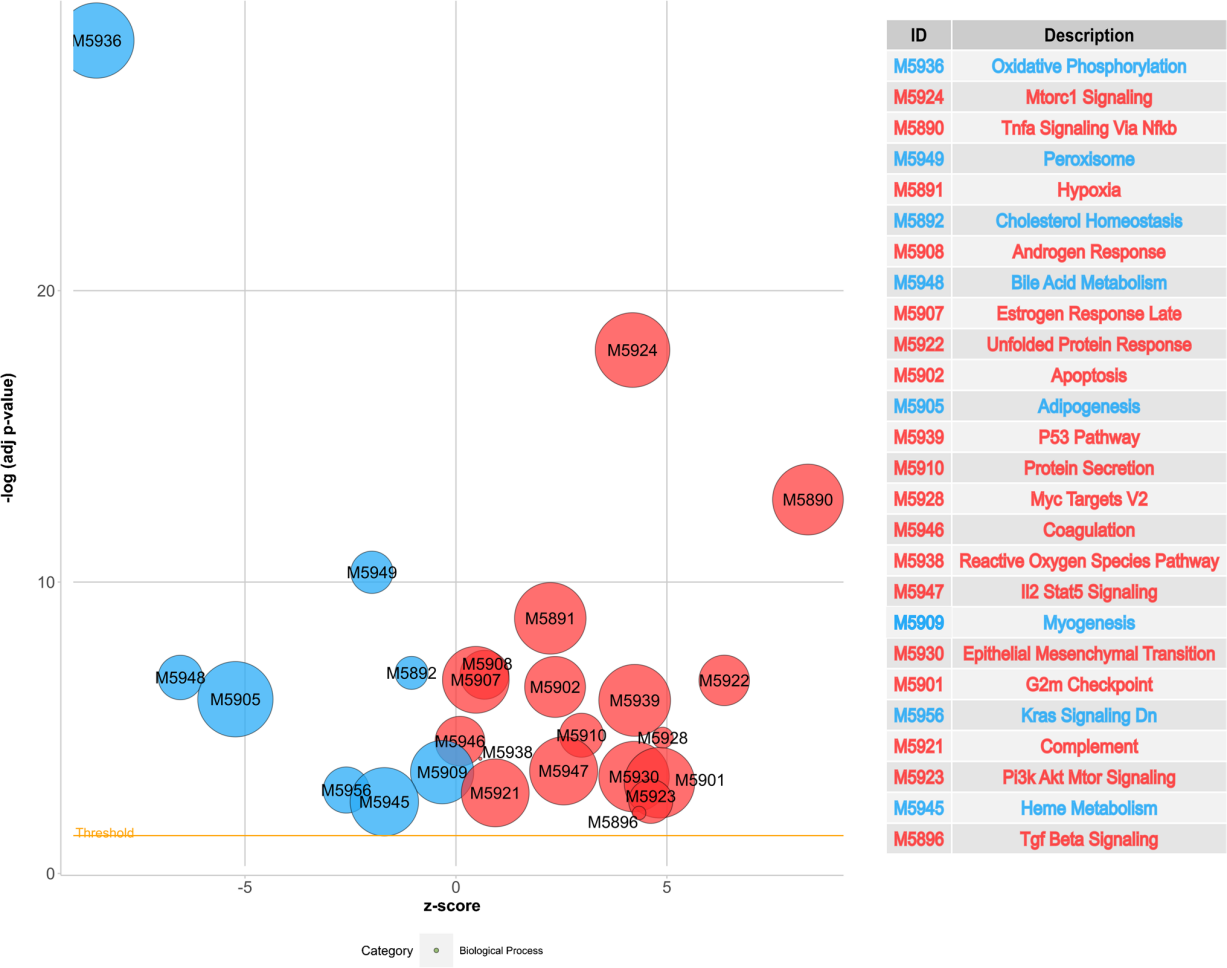
Transcriptomic analysis by next-generation sequencing (NGS) of control and RIAKI mouse kidneys. Bubble plots of transcriptomic analysis of 6016 regulated genes (adjusted *p*-value < 0.05). A negative *z*-score indicates that the majority of genes belonging to the pathway were downregulated (blue), and a positive *z*-score indicates that the majority of genes belonging to the pathway were upregulated (red). Gene set enrichment analysis revealed 26 significantly regulated pathways using the hallmarks annotation

The transcriptome of RIAKI was examined through next-generation sequencing (NGS), revealing 6016 regulated genes, with 2883 showing upregulation (refer to Table 1 for the top 10) and 3133 showing downregulation (refer to Table 2 for the top 10). Notably, the damage markers *Havcr1* (Kim-1) and *Lcn2* (Ngal) were among the top 10 upregulated genes (Table 1). Significantly, five of the top 10 markedly upregulated genes (*Spp1*, *Tnfrsf12a*, *Lgals3*, *Tsc22d1*, *Krt20*) play crucial roles in apoptosis. Pathway analysis utilizing Hallmarks annotation from MSigDB [25] identified regulation of 26 pathways in RIAKI (Fig. 3). These pathways include apoptotic pathways (“apoptosis,” “P53”), responses to cellular metabolic stress (“hypoxia,” “oxidative phosphorylation,” “Mtorc1 signaling,” “reactive oxygen species”), and those involved in the immune system (“Tnfα signaling via Nfκb,” “complement,” “Il2 Stat5 signaling”).

**Table 1:** Top 10 upregulated genes under RIAKI. Mice were subject to rhabdomyolysis for 24 h. Transcriptomic analysis served to detect changes in gene expression rate. Selected biological processes were identified using UniProt [58]

Gene	Name	Log2 fold change	*p*-value	Biological process
*Havcr1*	Hepatitis A virus cellular receptor 1	5,150	3,79E − 144	Phagocytosis, positive regulation of mast cell activation, response to lipopolysaccharide
*Plin2*	Perilipin-2	3,976	3,80E − 119	Cellular response to glucose starvation, lipid storage, long-chain fatty acid transport, positive regulation of sequestering of triglyceride
*Spp1*	Sphingosine-1-phosphate phosphatase 1	3,471	9,80E − 110	ER to Golgi ceramide transport, extrinsic and intrinsic apoptotic signaling pathways, phospholipid dephosphorylation
*Tnfrsf12a*	Tumor necrosis factor receptor superfamily member 12A	3,941	1,48E − 109	Angiogenesis, cell adhesion, cell differentiation, extrinsic apoptotic signaling pathway, positive regulation of apoptotic process, positive regulation of extrinsic apoptotic signaling pathway
*Lcn2*	Neutrophil gelatinase-associated lipocalin	4,900	1,98E − 93	Cellular response to hydrogen peroxide, extrinsic apoptotic signaling pathway, iron ion transmembrane transport, positive regulation of apoptotic process, positive regulation of endothelial cell migration, positive regulation of reactive oxygen species metabolic process
*Gsta1*	Glutathione S-transferase A1	5,896	4,15E − 87	Glutathione metabolic process, prostaglandin metabolic process
*Lgals3*	Galectin 3	3,146	7,52E − 83	Epithelial cell differentiation, macrophage chemotaxis, negative regulation of endocytosis, negative regulation of extrinsic apoptotic signaling pathway, regulation of extrinsic apoptotic signaling pathway via death domain receptors
*Tsc22d1*	TSC22 domain family protein 1	2,888	3,01E − 79	Negative regulation of apoptotic process, positive regulation of cell population proliferation, regulation of transcription by RNA polymerase II
*Mt2*	Metallothionein-2	3,555	5,11E − 78	Cellular response to copper and zinc ion, negative regulation of growth, nitric oxide mediated signal transduction
*Krt20*	Keratin, type I cytoskeletal 20	4,841	2,99E − 75	Apoptotic process, epithelial cell differentiation, intermediate filament organization

**Table 2:** Top 10 downregulated genes under RIAKI. Mice were subject to rhabdomyolysis for 24 h. Transcriptomic analysis served to detect changes in gene expression rate. Selected biological processes were identified using UniProt [58]

Gene	Name	Log2 fold change	*p*-value	Biological process
*Wfdc15b*	WAP four-disulfide core domain protein 15B	− 3,637	3,33E − 97	Innate immune response
*Gatm*	Glycine amidinotransferase, mitochondrial	− 3,509	5,26E − 88	Creatine metabolic process
*Slc7a13*	Solute carrier family 7 member 13	− 3,532	1,90E − 85	Amino acid transmembrane transport, aspartate transmembrane transport, L-cystine transport, L-glutamate transmembrane transport
*Egf*	Pro-epidermal growth factor	− 4,483	1,97E − 79	Angiogenesis, cell population proliferation, epithelial cell proliferation, ERBB2-EGFR signaling pathway, ERK1 and ERK2 cascade, negative regulation of secretion, positive regulation of epidermal growth factor-activated receptor activity, positive regulation of fibroblast proliferation, positive regulation of MAP kinase activity
*Nat8f1*	N-acetyltransferase 8 (GCN5-related) family member 1	− 3,138	5,46E − 77	
*Pah*	Phenylalanine-4-hydroxylase	− 3,087	6,41E − 76	L-phenylalanine catabolic process, protein hydroxylation
*Calb1*	Calbindin	− 2,875	3,71E − 75	Cellular response to organic substance
*Slc12a1*	Solute carrier family 12 member 1	− 4,310	1,20E − 73	Cell volume homeostasis, chloride ion homeostasis, potassium ion homeostasis, sodium ion homeostasis
*Akr1c21*	Aldo–keto reductase family 1 member C21	− 3,199	1,46E − 71	Steroid biosynthetic process, steroid metabolic process
*Ttc36*	Tetratricopeptide repeat protein 36	− 3,110	5,47E − 71	Cilium assembly, tyrosine metabolic process, negative regulation of proteasomal ubiquitin-dependent protein catabolic process

Overall, the gene expression pattern in RIAKI confirmed a pathway cascade known not only for RIAKI but also highly relevant in kidney pathology generally. One of the top-regulated genes, *Lgals3 alias* galectin-3 (Table 1), is associated with several of the RIAKI-regulated pathways including glycolysis/mTORC [7], ROS [16], inflammation [10], apoptosis [33], and epithelial to mesenchymal transition [61]. We, thus, focused on galectin-3, as the function of this factor is unknown in RIAKI.

### AGEs and AGE-receptors in RIAKI

**Fig. 4:**
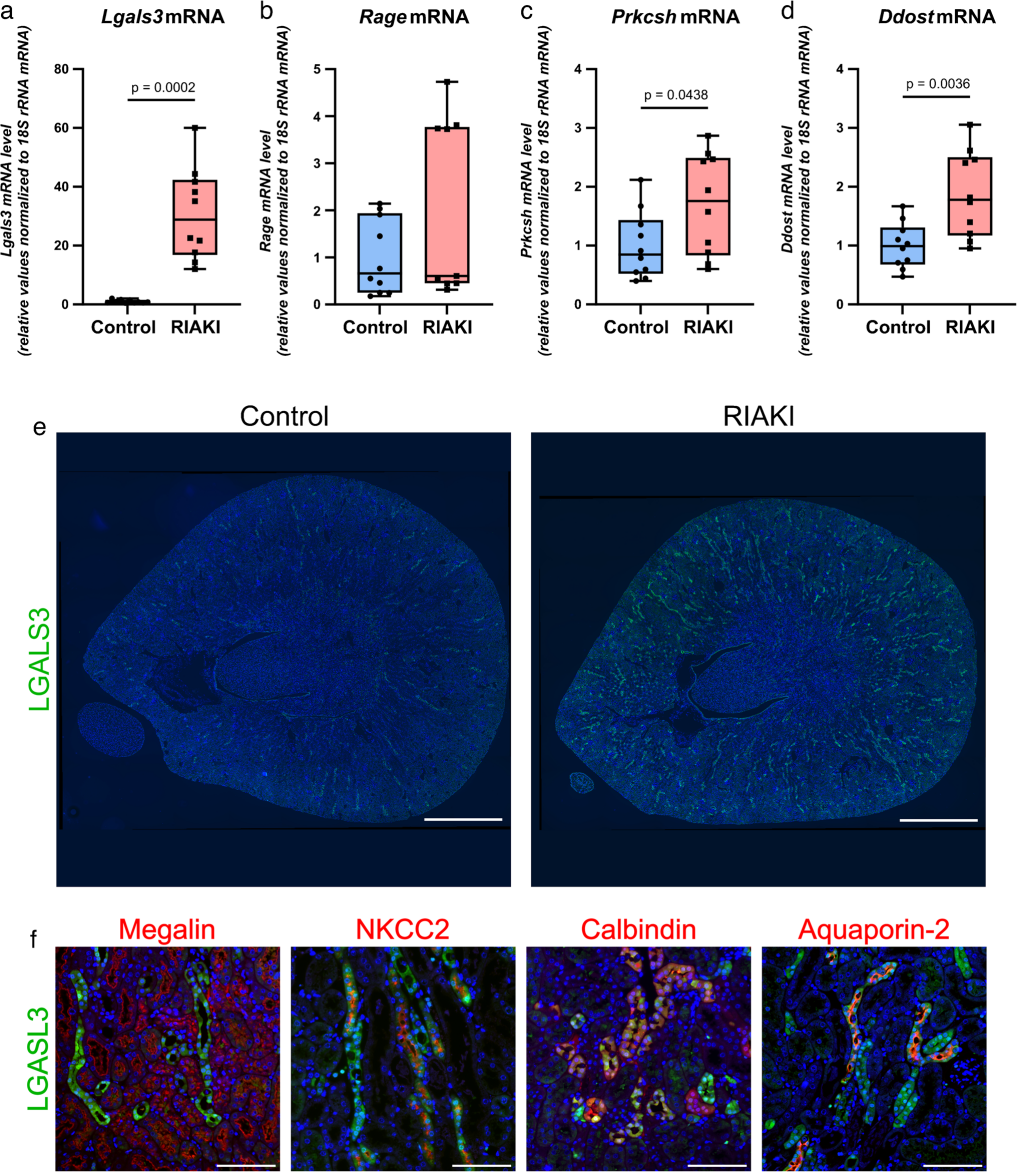
AGE-receptors in RIAKI. Mouse kidneys were analyzed 24 h after induction of RIAKI. **a**–**d** qPCR analysis for *Lgals3*, *Rage*, *Prkcsh*, and *Ddost*. Compared with controls, in RIAKI *Lgals3*, *Prkcsh*, and *Ddost* were significantly upregulated, while *Rage* was unchanged. Box plots show the median with lower and upper quartiles as a box. Whiskers show the minimum and maximum values. Dots represent single values. Statistical analysis was performed using either an unpaired *t* test (passed normality test with equal SD) or Welch’s *t* test (passed normality test with unequal SD). Adjusted *p*-values are shown. **e**, **f** Immunofluorescence for LGALS3 (green) and for nephron section markers (red) megalin (PT), NKCC2 (TAL), calbindin (DCT and CNT), and aquaporin-2 (CNT and CD), respectively. No LGALS3 was observed in controls, but in RIAKI, prominent signals appeared in all nephron segments, except for the PT. Scale bars: **e** 1000 µm; **f** 100 µm

**Fig. 5:**
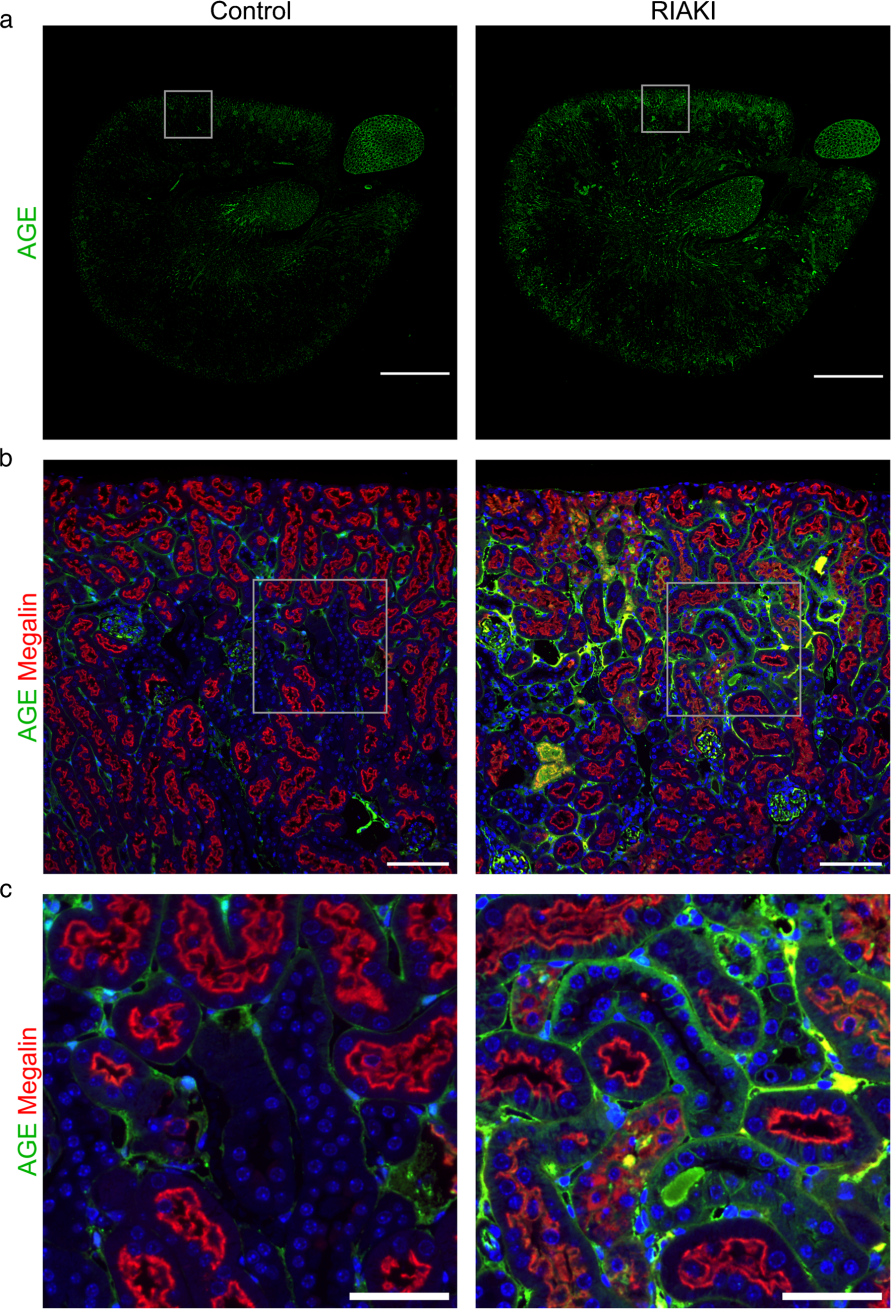
AGEs in RIAKI. Immunofluorescence on mouse kidneys 24 h after induction of RIAKI. **a**–**c** AGEs (green) and the proximal tubular marker megalin. In controls, rare AGE signals appear in the interstitium and the basolateral portion of distal tubules. In RIAKI, predominantly in the cortex, the basolateral portion of distal tubules and the interstitium are strongly positive for AGE. Scale bars: **a** 1000 µm; **b** 100 µm; **c** 50 µm

Upregulation of galectin-3 mRNA (*Lgals3*) in RIAKI was confirmed via qPCR (Fig. 4a). Galectin-3, known as an AGE receptor, is recognized for its inhibitory effect on the apoptosis signaling pathway [33]. Given the upregulation of the “hypoxia” pathway and downregulation of “oxidative phosphorylation” in RIAKI (Fig. 3), we proposed an increased rate of anaerobic metabolism. This metabolic shift generates methylglyoxal, the main source of AGEs, which necessitates glutathione as a co-factor [54]. Thus, we suggested that LGALS3 and AGEs might play pivotal roles in RIAKI, prompting a closer examination of their presence in the tubular system. In addition to LGALS3, common AGE receptors include RAGE, DDOST, and PRKCSH [21]. While *Rage* showed no differences in gene expression or protein levels compared to control animals (Fig. 4b and Supplementary Fig. 2, respectively), *Ddost* and *Prkcsh* mRNA levels were significantly upregulated in RIAKI mice (Fig. 4c, d). Nevertheless, *Lgals3* exhibited the most prominent alteration. Further immunofluorescence studies revealed an increased presence of LGALS3 protein in RIAKI (Fig. 4e). Co-expression analysis using tubular segment-specific markers showed that LGALS3 expression was confined to distal nephron segments, excluding the PT (Fig. 4f). To further investigate the role of AGEs in RIAKI, glycated molecules were analyzed using immunofluorescence and western blotting (Fig. 5 and Supplementary Fig. 3, respectively). In RIAKI, AGEs were significantly elevated in the cortex interstitium, distal tubular segments, and the glomerular compartment (Fig. 5a–c).

In summary, our findings suggest that AGE signaling is crucial in RIAKI. LGALS3 is strongly upregulated in tubular cells of the distal nephron, which are less impaired in RIAKI. Thus, we propose that upregulation of LGALS3 in distal tubules may serve as a potential defense against the toxic effects of AGEs and subsequent apoptosis, as observed in proximal tubules. To test this hypothesis, in vitro experiments were conducted to examine the influence of AGEs on distal convoluted tubule (DCT) cells with and without *Lgals3* expression.

### LGALS3 protects against AGE-induced apoptosis in vitro

**Fig. 6:**
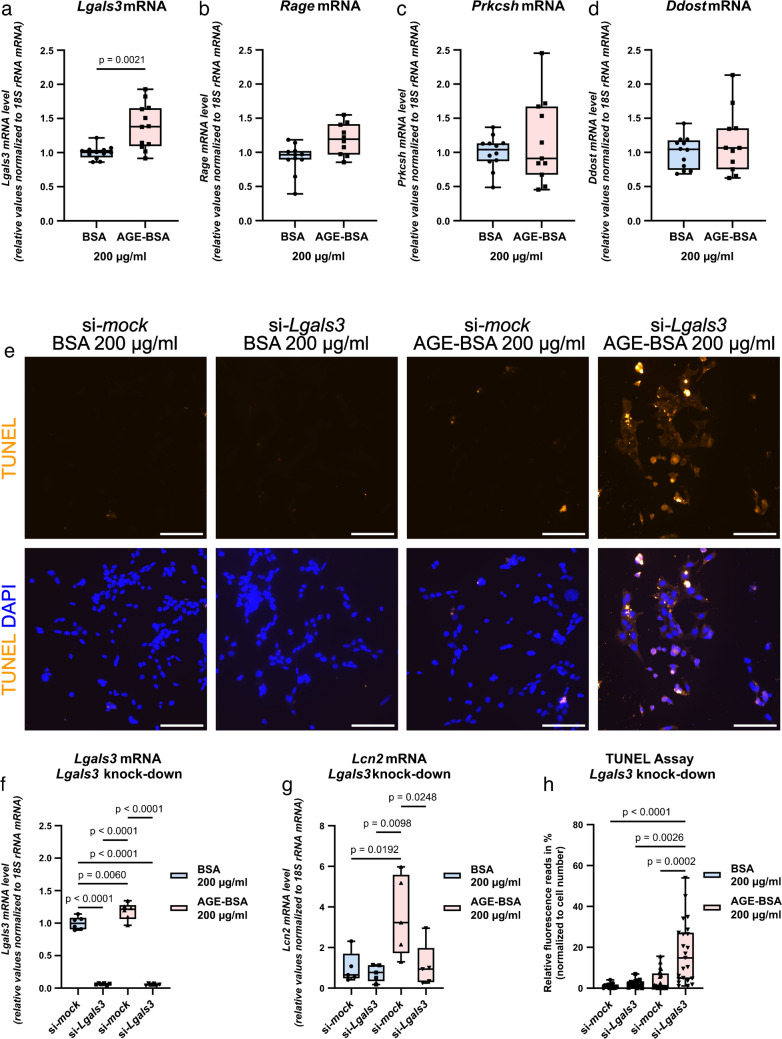
Absence of *Lgals3* causes increased AGE-mediated apoptosis in vitro. DCT cells treated for 24 h (**a**–**d**) or 48 h (**e**–**h**) with either BSA (control), AGE-BSA, or AGE-BSA-*Lgals3*-siRNA. **a**–**e**: qPCR analysis of AGE receptors *Lgals3*, *Rage*, *Prkcsh*, and *Ddost*. *Lgasl3* is significantly upregulated by AGE-BSA. **e**, **h**: Immunofluorescence for apoptosis (TUNEL assay). *Lgals3*-depleted cells show enhanced AGE-induced apoptosis. **f**: *Lgals3*-siRNA treatment downregulates *Lgals-3* mRNA by over 90%. **g**: qPCR analysis of *Lcn2* indicated upregulation following AGE-BSA treatment that is abolished by *Lgals3* knockdown. Box plots show the median with lower and upper quartile as box. Whiskers show the minimum and maximum values. Dots represent single values. Statistical analysis was performed with either an unpaired *t* test (passed normality test with equal SD) or Kolmogorov–Smirnov test (did not pass normality test with unequal SD) in **a**–**d**, or with help of ordinary one-way ANOVA followed by Tukey’s post-hoc test in **f**–**h**. Adjusted *p*-values are shown. Scale bar: 100 µm

DCT cells were stimulated with AGE-modified bovine serum albumin (AGE-BSA), a common inducer of AGE signaling in vitro [6, 51]. Remarkably, AGE-BSA significantly increased *Lgals3* mRNA levels (Fig. 6a), while mRNA levels of *Rage*, *Prkcsh*, or *Ddost* remained unchanged (Fig. 6b–d).

Subsequent inhibition of AGE-mediated upregulation of *Lgals3* was achieved through RNAi technique (Fig. 6e–h). In DCT cells, neither 48 h of *Lgals3* knockdown alone nor exposure to AGE-BSA induced apoptosis, as detected by TUNEL assay (Fig. 6e, h). Notably, when cells were treated with AGE-BSA, *Lgals3* knockdown resulted in a significantly higher number of apoptotic cells (Fig. 6e, h). Furthermore, AGE-BSA caused upregulation of the injury marker *Lcn2* (*alias Ngal*) that was abolished by *Lgals3* (Fig. 6g). Thus, our findings suggest a protective role of galectin-3 against AGE-mediated apoptosis.

## Discussion

Our study has two main findings: First, in vivo, AGE receptor LGALS3 is upregulated in nephron segments protected from RIAKI; Second, in vitro, LGALS3 downregulation exacerbates AGE-induced cell injury.

AGEs/AGE receptor interaction was predicted to play a role in the pathophysiology of RIAKI according to our transcriptomic analyses. This prompted us to conduct further morphological and mechanistic studies. Indeed, the major site of acute cell damage, the proximal tubule, was subject to AGEs-induced stress with no LGALS3 expression. By contrast, the relatively well-preserved distal nephron was stressed as well but may have mounted a protective response via LGALS3. This hypothesis is backed by further studies conducted in vitro. Therefore, LGALS3 is a promising renoprotective target, specifically for RIAKI.

Rhabdomyolysis occurs after acute skeletal muscle destruction and rapidly causes multiple organ failures, most notably AKI [19]. RIAKI affects up to 46% of hospitalized patients and up to 80% of patients in intensive care units [4, 19, 28], with mortality rates above 15% [35]. Although the relevance of RIAKI is doubtless, therapy options are limited. Recent advances include inhibition of myoglobin endocytosis, prevention and treatment of oxidative damage, and immune cell targets [19]. With the help of transcriptomic profiling and gene set enrichment analysis, we confirmed established contributors of RIAKI, such as metabolic dysregulation, hypoxia, ROS, and inflammation. Nevertheless, we also observed an unexpectedly strong upregulation of *Lgals3* in RIAKI. LGALS3, *alias* galectin-3 (Gal3) is expressed in diverse tissues and regulates cell growth, proliferation, differentiation, inflammation, phagocytosis, exocytosis, and fibrosis [12]. Functionally, LGALS3 has been mainly described to induce pro-inflammatory and pro-fibrotic responses [60]. Moreover, LGALS3 has been implicated in the development of both AKI and chronic kidney disease (CKD) [60]. Circulating LGALS3 is associated with loss of kidney function due to impaired urinary clearance [62]. Although LGALS3 can be secreted into the circulation, it is predominantly located in the cytoplasm but can also shuttle into the nucleus [5]. Interestingly, intracellular LGALS3 is important for cell survival due to its ability to block the intrinsic apoptotic pathway, while intra-nuclear LGALS3 promotes cell proliferation [5, 9]. Thus, intracellularly, the impact of LGALS3 is anti-apoptotic, whereas when situated extracellularly, it shows pro-apoptotic characteristics [29]. Mechanistically, LGALS3 showed a direct influence on anti-apoptotic proteins belonging to the BCL-2 family [29, 34]. Activation of these proteins hinders the release of cytochrome c from mitochondria, consequently halting the process of apoptosis [11]. Moreover, *Lgals3* knockdown in DCT cells prevented AGE-BSA mediated elevation of *Lcn2* (NGAL). Although NGAL is used as a kidney injury marker, it has several protective cellular functions: NGAL suppresses extracellular iron-induced injury [27], acts anti-inflammatory by the inhibition of NfκB [63], and stimulates proliferation and differentiation [46, 50]. LGALS3, thus, seems to be crucial to activate protective factors in distal tubules.

Moreover, LGALS3 knockout mice developed significant glomerular sclerosis [22], showed greater susceptibility to AGE-induced renal disease, increased levels of AGE and signaling, and altered patterns of AGE-receptors [22]. Supporting this, Pugliese et al*.* showed that LGALS3-regulated pathways activate protection against AGE-induced tissue injury [43]. These data suggest that LGALS3 plays a crucial role as an AGE receptor and mediates protection against AGE-dependent damage [5]. We subsequently confirmed an elevated abundance of AGEs in RIAKI. In healthy individuals, AGEs are filtered by the glomeruli and absorbed in the proximal tubules, where they are metabolized by the proximal tubule cells. Excessive uptake of AGEs can lead to dysfunction of the tubular lysosomes in these cells [13]. In vitro, the knockdown of *Lgals3* abolished the ability of cells to prevent AGE-induced apoptosis, confirming the protecting role of cytoplasmic LGALS3. However, the source and individual activities of different AGEs in RIAKI have to be identified in further studies. At least, the presence of AGEs in the glomerular compartment supports the idea that, since rhabdomyolysis also affects other organs, glomerular AGEs could arise from elevated systemic AGEs originating from sources outside the kidney. Due to the toxic properties of AGEs [57], their occurrence fills a gap in our current understanding of RIAKI pathology.

It remains to be an open issue why only distal tubules showed elevated LGALS3 expression, thus protection against AGE-mediated cell stress, while proximal tubules were heavily injured in RIAKI. Nishiyama et al. investigated the impact of LGALS3 on cell injury as well as cell regeneration in rats exposed to ischemic and toxic acute renal failure (ARF) [36]. The authors showed raised LGALS3 levels as early as 2 h after injury in proximal tubules. This suggests that the PT has the potential to upregulate LGALS3. Supporting, single-cell sequencing (scRNAseq) data from mouse kidneys that underwent ischemia–reperfusion injury (IRI) indicated elevated *Lgals3* mRNA in the entire nephron, including the PT (http://www.humphreyslab.com/SingleCell/search.php) [23]. Moreover, scRNAseq data from human biopsies of control *vs*. AKI patients show significantly upregulated *LGALS3* mRNA in proximal tubules, thick ascending limb, distal convoluted tubules, connecting tubules, collecting duct principal cells, and endothelial cells (https://shiny.mdc-berlin.de/humAKI/) [20]. Obviously, in RIAKI, protective LGALS3 activation in proximal tubules fails and might represent a mechanistic difference to other forms of AKI that warrants further investigations.

In conclusion, this study adds mechanistic details to the pathology of RIAKI. Our findings highlight AGEs and their receptors, particularly LGALS3, in influencing renal injury, which arises from a multifaceted interaction involving glucose metabolism, oxidative stress, inflammation, and apoptosis. Further investigations are needed to elucidate potential therapeutic strategies targeting AGE-receptor interactions to alleviate renal damage.

## Supplementary Information

Below is the link to the electronic supplementary material.

Supplementary file1 (PDF 20814 KB)

## Data Availability

NGS data shown in this study are provided via GEO accession no. GSE264651 at https://www.ncbi.nlm.nih.gov/geo/query/acc.cgi?acc=GSE264651
