# Supplementary material for: Galectin-3 protects distal convoluted tubules in rhabdomyolysis-induced kidney injury
Source: Pflugers Arch. 2024 Jul 23;476(10):1571–85. doi: 10.1007/s00424-024-02987-0 (PMC11381487; doi:10.1007/s00424-024-02987-0)
Supplement: Supplementary file 1 — Supplementary file1 (PDF 20814 KB) [file 424_2024_2987_MOESM1_ESM.pdf]

Supplementary information

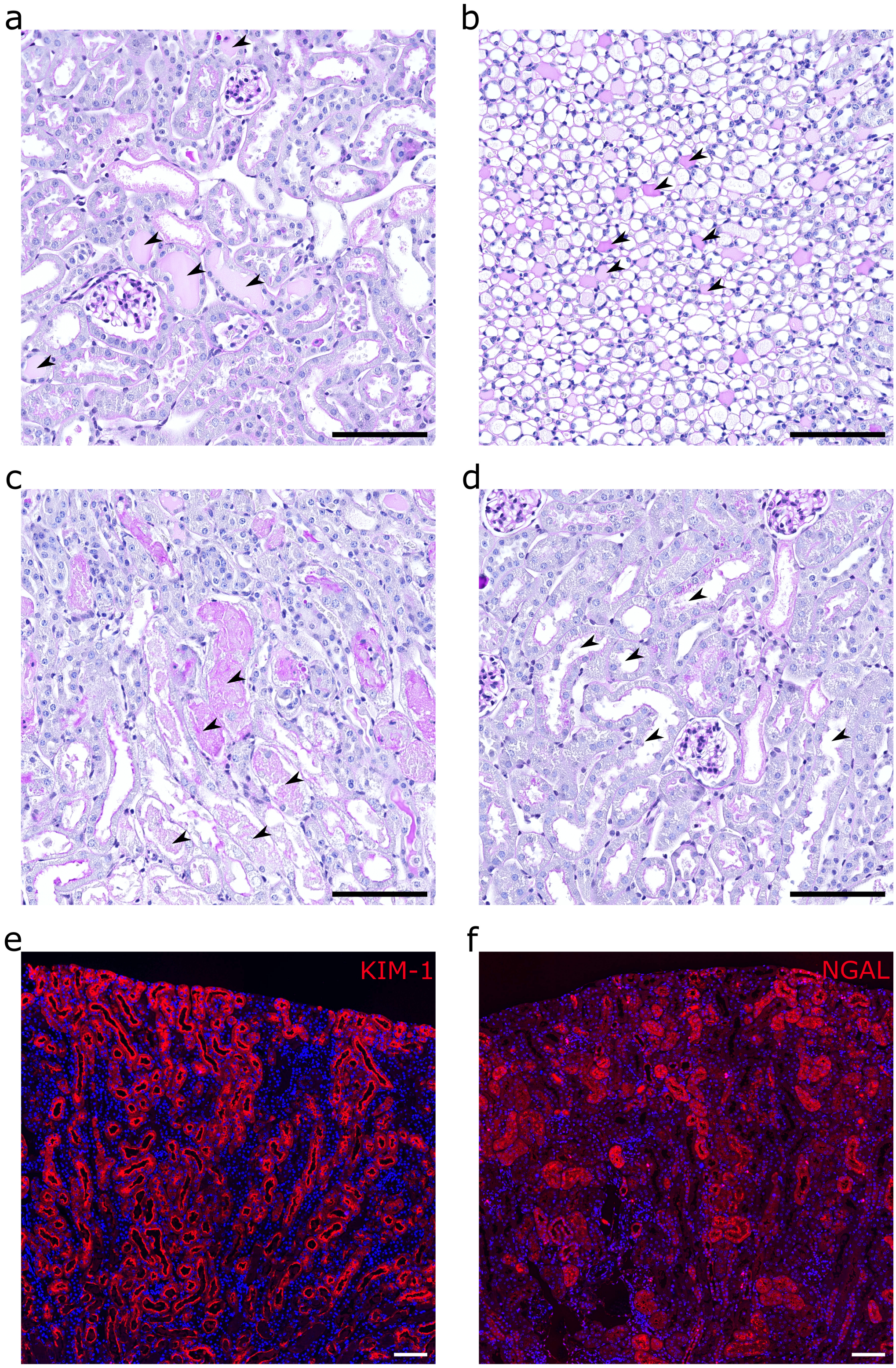

Supplementary Fig. 1 Pathological changes and elevated kidney injury markers following RIAKI. **a-d:** Periodic Acid-Schiff staining of mouse kidneys following 24 h RIAKI **a, b**: Arrow heads indicate tubular obstruction/ intratubular cast formation in the cortex (**a**) and in the papilla (**b**). **c**: Arrow heads indicate tubular necrosis. **d**: Arrow heads indicate loss of brush border in proximal tubules. **e, f**: Immunofluorescence staining of the kidney injury markers KIM-1 (**e**) and NGAL (**f**) in RIAKI kidneys. Scale bar: 100 µm

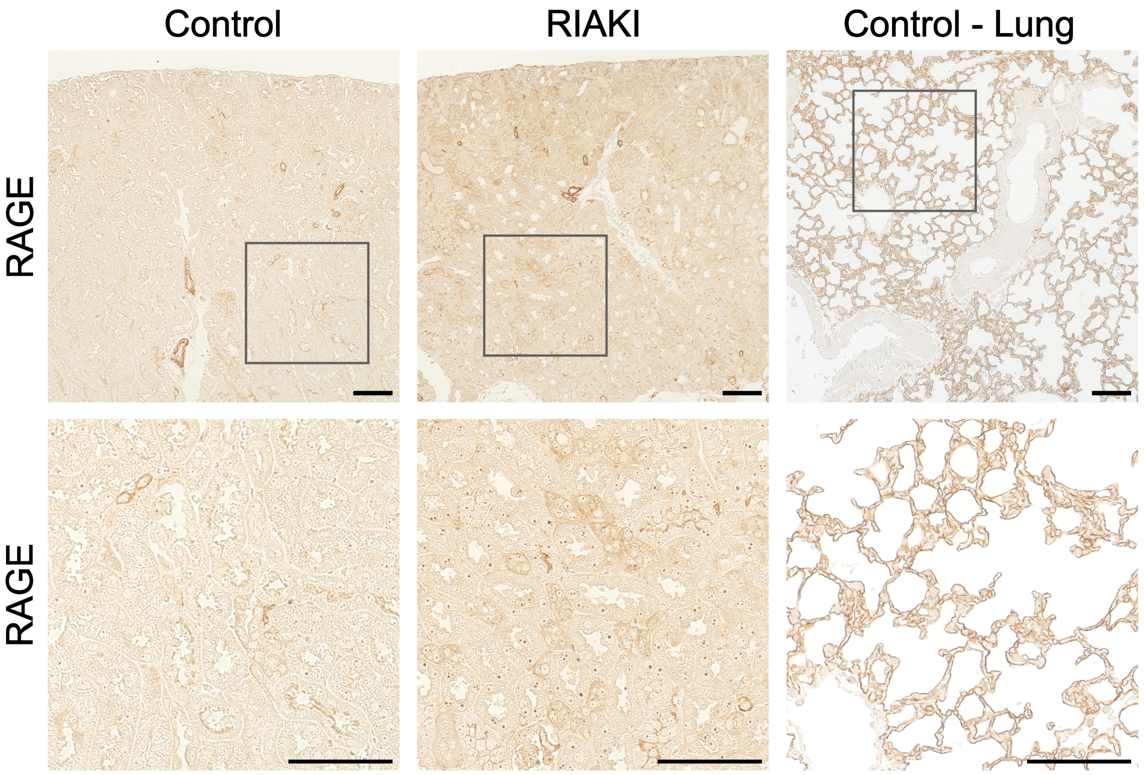

Supplementary Fig. 2 Unchanged renal RAGE expression in RIAKI. Immunohistochemistry of RAGE on control and RIAKI mouse kidneys with mouse lung as positive control. No alteration of RAGE expression was observed following RIAKI. Scale bar: 100 µm.

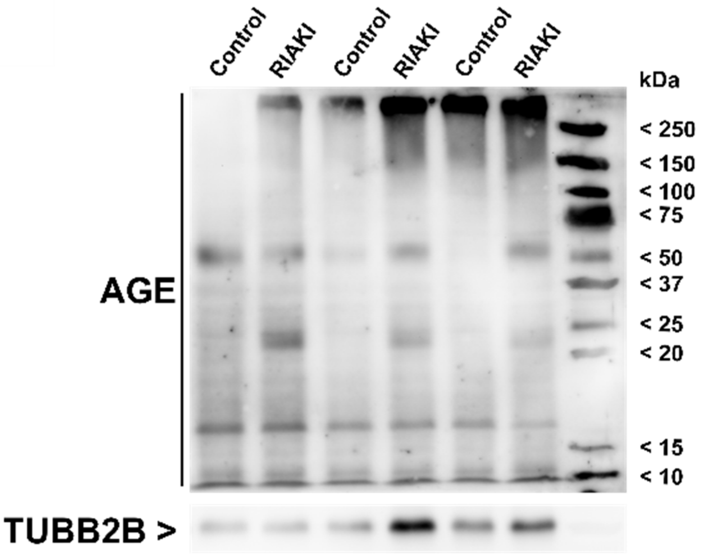

Supplementary Fig. 3 Renal AGE accumulation in RIAKI. Western Blot to detect AGEs in control and RIAKI mouse kidneys. RIAKI kidneys show the occurrence of new AGE-positive protein bands.

Supplementary Table 1 List of primers used for qPCR.

Target Gene (mouse)	Primer Sequence (5´ to 3´)
Havcr1	Forward: TCAGGGTCTCCTTCACAGCAG
	Reverse: TGACCCACCACCCCCTTTAC
Lcn2	Forward: CCAGACTTCCGGAGCGATCA
	Reverse: GTGGTGGCCACTTGCACATT
Rage	Forward: CACCCAAGGAGGAACCCATC
	Reverse: GATGCTGACAGGAGGGCTTT
Lgals3	Forward: GCACAGAGAGCACTACCCAG
	Reverse: TTGGGTTTCACTGTGCCCAT
Ddost	Forward: TCCCTACTATGCCAGTGCCT
	Reverse: CCGACCGATCCATTCGTGAT
Prkcsh	Forward: AGAGAGGCCAGAGAAGGAGG
	Reverse: GGAGGTGGTGTCAGTCTGTG
18S rRNA	Forward: GATCAAAACCAACCCGGTCA
	Reverse: CCGTTTCTCAGGCTCCCTCT

Supplementary Table 2 Antibodies used in this study.

Antibody target	Host	Supplier/ RRID
Primary antibodies		
**active Caspase-3**	polyclonal rabbit	#ab2302; abcam, UK; RRID:AB_302962
**AGE**	polyclonal rabbit	#ab23722, abcam, UK; RRID:AB_447638
**Aquaporin-2**	polyclonal rat	#20102rs; BiCell Scientific, USA; RRID:AB_2910118
**Aquaporin-2**	polyclonal rabbit	#sc-9882; Santa Cruz Biotechnology, USA; RRID:AB_2289903
**Calbindin**	monoclonal mouse	#C9848, Sigma-Aldrich, USA; RRID:AB_476894
**Galectin-3**	monoclonal rat	LGALS3; #14-5301-82; Invitrogen, USA; RRID:AB_837132
**KIM-1**	polyclonal goat	#AF1817, R and D Systems, USA; RRID:AB_2116446
**Megalin**	monoclonal mouse	LRP2; #ab184676, abcam, UK; RRID:AB_2910117
**NKCC2**	polyclonal rabbit	#LS-C313275, Lifespan Biosciences, USA; RRID:AB_2910114
**RAGE**	polyclonal rabbit	#PA1-075, Invitrogen, USA RRID:AB_2269243
**TUBB2B**	polyclonal rabbit	#TA337744, OriGene Technologies, USA; RRID:AB_2910113
Secondary antibodies		
**anti-goat Alexa Fluor 488**	donkey	#705-545-147; Jackson Immuno Research Labs; RRID:AB_2336933
**anti-goat Alexa Fluor 594**	donkey	#705-585-147; Jackson Immuno Research Labs; RRID:AB_2340433
**anti-mouse Cy3**	donkey	#715-165-150; Jackson Immuno Research Labs, RRID: AB_2340813
**anti-rabbit Alexa Fluor 488**	donkey	#711-545-152; Jackson Immuno Research Labs; RRID:AB_2313584
**anti-rabbit Cy3**	goat	#111-165-003; Jackson Immuno Research Labs, RRID: AB_2338000
**anti-rabbit IgG-HRP**	goat	#sc-2030, Santa Cruz Biotechnology, USA; RRID:AB_631747
**anti-rat Cy3**	donkey	#712-165-150; Jackson Immuno Research Labs, RRID:AB_2340666
**anti-rat IgG-HRP**	goat	#sc-2006, Santa Cruz Biotechnology, USA; RRID:AB_1125219
